# Do cancer patients' psychosocial outcomes and perceptions of quality of care vary across radiation oncology treatment centres?

**DOI:** 10.1111/j.1365-2354.2011.01299.x

**Published:** 2012-05

**Authors:** M CAREY, C PAUL, L MACKENZIE, R SANSON-FISHER, E CAMERON

**Affiliations:** Faculty of Health, University of NewcastleCallaghan, NSW, Australia

**Keywords:** cancer, oncology, psychosocial, anxiety, depression, patient-centred care

## Abstract

This study aimed to explore whether rates of depression, and anxiety and patient views about quality of patient-centred care varied across four metropolitan radiation therapy treatment centres in Sydney, Australia. Participants were radiation therapy outpatients, aged 18 or older and English-speaking. Participants completed a brief survey by touch screen computer while waiting for their radiation therapy treatment appointment. For eight indicators of patient-centred care, participants were asked to indicate whether their well-being would have been improved by better care related to the indicator. Participants also completed the Hospital Anxiety and Depression Scale. No differences between treatment centres were found for rates of anxiety and depression, or for the mean number of domains of care endorsed as needing improvement (indicated by agreeing or strongly agreeing that their well-being would have been improved by better care). The lack of variance in these outcomes may reflect that variation in treatment centre characteristics does not influence psychosocial outcomes and patient views of their care. Alternatively, it may suggest that the characteristics of the four treatment centres which participated in the present study were too similar for differences in patient outcomes to be observed.

## INTRODUCTION

Patient-centred care has been broadly defined as care which is responsive to the needs, values and preferences of patients (Gerteis *et al*. 1993; Institute of Medicine 2001). Domains commonly identified as important to patient-centred care include: (1) physical comfort; (2) emotional support; (3) respect for patient preferences; (4) integration and coordination of care; (5) information and education; and (6) involvement of family and friends (Institute of Medicine 2001). There is increasing acceptance of this as a key dimension of quality of care, with several government and non-government organisations developing policy documents and position papers on patient-centred care (Luxford *et al*. 2010). Because assessment of the quality of patient-centred care requires a judgment as to how well patient's needs and preferences were met by the care provided, patients play an essential role in this evaluation process (Stewart 2001; Coulter 2006). Several countries have introduced patient surveys to evaluate quality in this domain. The National Health Service in the UK conducts regular surveys of patients to monitor trends in patients' views about their care over time. One of these surveys specifically focussed on the experiences of people with cancer (Airey *et al*. 2002). Similarly, in Canada and Australia patient experience surveys have been implemented to examine cancer patients' views about the quality of their care (Watson *et al*. 2007; Heading *et al*. 2008). While results of these surveys generally indicate that patients perceive that their care is good across most domains, the need for improvements in information provision and emotional support are commonly identified (Watson *et al*. 2007; Heading *et al*. 2008).

Descriptive studies suggest that there is an association between patient-centred care and outcomes such as better physical health (Fremont *et al*. 2001), reductions in 1-year mortality among cardiovascular disease patients (Meterko *et al*. 2010) and receipt of preventive care by Veteran's Health Administration clients (Flach *et al*. 2004). Randomised controlled trials indicate that interventions may improve delivery of patient-centred care; however, the extent which these influence patient health behaviours and other health outcomes is not clear (Lewin *et al*. 2001). In particular, there is little research on this topic of relevance to cancer patients. It is plausible, for example, that hospitals which deliver high-quality patient-centred care may have patients who report lower rates of anxiety, depression, unmet needs and better symptom control than other hospitals. This may be because such needs are anticipated, planned for and addressed in a timely manner within such an environment. Despite this, our recent review failed to identify any studies which examined whether variation in systems, structures or other processes of care between treatment centres influence cancer patients' psychosocial outcomes (Carey *et al*. 2011). This study aimed to examine: (1) whether there is variation between cancer treatment centres in number of domains of care which cancer patients report as needing improvement; and (2) whether there is variation between cancer treatment centres in the proportion of patients reporting clinically significant anxiety and depression.

## METHODS

### Setting

A convenience sample of four radiation oncology treatment centres in metropolitan New South Wales, Australia was recruited. All centres were attached to public hospitals, and had two to four linear accelerators in use. Average patient throughput varied between approximately 60 and 140 patients per day. Ethics approval for the study was gained from the University of Newcastle and Cancer Institute of New South Wales Human Research Ethics Committees.

### Participants

Eligible patients were diagnosed with cancer, presenting for a radiation therapy treatment appointment, aged 18 years or older and English-speaking. Participants who were presenting for their first clinic appointment and those who were judged too sick to participate by clinic staff were excluded.

### Procedure

A research assistant assessed eligibility for the study and sought informed consent from eligible patients. Consenting patients were asked to complete a 10- to 15-min touch screen computer survey. Questions were presented on screen and participants were instructed to ‘touch’ the response on screen which corresponded to their answer. Results relating to which domains are most frequently endorsed as requiring improvement and disease and socio-demographic variables associated with patients indicating that none of the eight domains of care require improvement will be reported elsewhere.

### Measures

#### Demographic questions

Respondents were asked to indicate their age, gender, postcode, country of birth, health insurance and living arrangements.

#### Medical variables

Type of cancer, number of weeks' since diagnosis and whether the person was currently having treatment were assessed by self-report.

#### Quality of care

An introductory screen to the quality of care questions read ‘Cancer patients have suggested that improvements in some areas of care may improve their well-being. We would like you to tell us which of the following aspects of care (if any) could have been improved since you were diagnosed with cancer. Your answers will be confidential. Your answers may help us to identify areas where care may be improved’. Perceived quality of care was assessed using the following question stem: ‘During my cancer care, my well-being would have been greatly improved by: …’. Response options were presented on a 4-point Likert scale (1 = strongly disagree; 4 = strongly agree). Eight domains of quality of care were assessed with a single question for each: (1) better management of my physical symptoms; (2) better information and communication about my cancer and care; (3) better emotional and/or spiritual support; (4) better services, information and support for my friends/family; (5) better staff approachability and respect for me; (6) getting better access to the care I need when required; (7) better services/support to cope with changes to my relationships; and (8) better services/advice to assist me with practical concerns. An additional sentence describing each of the domains and examples of what might fit under each domain was presented below each item. Items were derived from the Institute of Medicine criteria for delivery of patient-centred care (Institute of Medicine 2001). Domains which were endorsed as needing improvement were summed for each participant. Items were pilot-tested with 67 participants. Minor changes to wording were made to ensure the items were perceived as relevant and easy to understand by the target population.

*The Hospital Anxiety and Depression Scale* (HADS) was used to assess clinically significant anxiety and depression. A recent review has recommended the HADS as the instrument of choice for assessing psychological morbidity among those with cancer (Luckett *et al*. 2010). The HADS meets psychometric criteria for internal consistency (Lloyd-Williams *et al*. 2001), construct validity (Moorey *et al*. 1991) and discriminant validity (Walker *et al*. 2007). While findings are mixed with respect to the optimal cut-off score to define caseness, a score of 8 has been recommended by a previous review (Bjelland *et al*. 2002) to achieve an optimal balance between sensitivity and specificity (Love *et al*. 2002). A subscale score of 8 was used in the current study to indicate possible anxiety and depression on the anxiety and depression scales respectively.

## RESULTS

### Consent rates

A total of 641 participants were assessed for eligibility. Of these, 132 were ineligible primarily because they were non-English speakers or it was their first visit to the treatment centre. Of the 509 eligible patients, 431 consented to participate. Among those consenting to the study, 346 completed the survey, 13 withdrew after starting the survey and 72 were unable to complete the survey due to time constraints. This gave a consent rate of 85% and completion rate of 80%. There was little difference between the four treatment centres in terms of the demographic and disease characteristics of participants (Table 1). The age of participants and the types of cancers seen at each treatment centre were significantly different.

**Table 1:** Demographic and disease characteristics of participants

Characteristic	Clinic 1	Clinic 2	Clinic 3	Clinic 4	Test of independence
Male, *n* (%)	50 (50)	48 (49)	39 (55)	40 (52)	χ^2^ (3) = 0.643, *P*= 0.890
Mean age (SD)	58.1 (15.3)	63.6 (12.5)	60.1 (11.8)	63.3 (13.7)	*F*(3,342) = 3.476, *P*= 0.016
Private health insurance, *n* (%)	51 (50)	65 (67)	36 (54)	45 (62)	χ^2^ (3) = 6.468, *P*= 0.092
Australian born, *n* (%)	68 (67)	64 (66)	44 (62)	55 (71)	χ^2^ (3) = 1.531, *P*= 0.677
Living alone, *n* (%)	21 (21)	19 (20)	11 (15)	21 (27)	χ^2^ (3) = 3.258, *P*= 0.354
Currently receiving treatment, *n* (%)	96 (95)	93 (96)	70 (99)	77 (100)	χ^2^ (3) = 4.901, *P*= 0.185
Perceive curative intent of treatment, *n* (%)	40 (42)	44 (47)	35 (50)	36 (47)	χ^2^ (3) = 1.256, *P*= 0.742
Second diagnosis and/or recurrence, *n* (%)	26 (26)	31 (32)	24 (34)	15 (19)	χ^2^ (3) = 4.984, *P*= 0.173
Mean weeks since diagnosis (SD)	85 (163.6)	113 (230.2)	77 (131.3)	72 (168.5)	*F*(3,342) = 0.917, *P*= 0.433
Cancer type, *n* (%)					χ^2^ (18) = 33.951, *P*= 0.013
Brain	8 (8)	2 (2)	1 (1)	4 (5)	
Breast	26 (26)	35 (36)	12 (17)	20 (26)	
Colorectal	6 (6)	7 (7)	6 (8)	1 (1)	
Head and neck	9 (9)	5 (5)	9 (13)	10 (13)	
Lung	4 (4)	2 (2)	7 (10)	2 (3)	
Prostate	15 (15)	21 (22)	14 (20)	24 (31)	
Other	33 (33)	25 (26)	22 (31)	16 (21)	

### Perceived quality of care

The number of domains perceived by individual participants as needing improvement ranged from 0 to the maximum of 8 with an overall mean of 1.56 (SD = 2.42). There was no significant difference between the mean number of domains identified as needing improvement at each treatment centre (Kruskal Wallis non-parametric test, χ^2^ (3) = 5.811, *P*= 0.121) (Table 2). Furthermore, across each of the eight domains separately, there was no significant difference between treatment centres in the number of participants endorsing each domain. The mean number of domains reported as needing improvement for each treatment centre is presented in Table 2.

**Table 2:** Mean number of domains of care in which better care would have improved patient well-being (by treatment centre)

Clinic	Number of respondents	Mean number of domains needing improvement (SD)
1	101	1.76 (2.42)
2	97	1.16 (2.21)
3	71	1.73 (2.69)
4	77	1.64 (2.38)
Total	346	1.56 (2.42)

Using a HADS cut-off of 8, 28% of patients were classified as having possible anxiety and 17% as having possible depression (Table 3). There was no significant difference between the treatment centres in the number of anxious (χ^2^ (3) = 2.717, *P*= 0.437) or depressed participants (χ^2^ (3) = 1.327, *P*= 0.723). Rates of possible anxiety and depression are shown for each treatment centre in Table 3.

**Table 3:** Number and percentage of patients with possible anxiety and depression (by treatment centre)

Clinic	Number of respondents	Clinically significant anxiety*, *n* (%)	Clinically significant depression*, *n* (%)
1	100	24 (24)	18 (18)
2	95	24 (25)	16 (17)
3	71	24 (34)	14 (20)
4	77	24 (31)	10 (13)
Total	343	96 (28)	58 (17)

* Hospital Anxiety and Depression subscales score ≥8.

## DISCUSSION

Previous studies with cancer patients have reported rates of possible anxiety between 27% (Moorey *et al*. 1991) and 48% (Stark *et al*. 2002) and rates of possible depression ranging between 9% (Moorey *et al*. 1991) and 20% (Carroll *et al*. 1993) using a HADS threshold of 8. Therefore, the results of the present study fell within lower end of the prevalence range for anxiety reported by previous studies. Results for depression fell within the middle of the prevalence range reported by studies.

The measure of quality of care used in the present study was new and thus precluded comparison with past studies. Respondents were asked to indicate if better care in each domain would have improved their well-being. Previous research on quality of life has used patient judgements to determine the clinical significance of changes in quality of life over time (Osoba *et al*. 1998). While the average number of care domains reported as needing improvement was only one or two, it is possible that indicating the need for improvements in even a single domain may be clinically important.

Contrary to expectations, neither rates of anxiety or depression nor the mean number of care domains reported as needing improvement varied significantly among the four hospitals. The data therefore suggest that while individual variations are evident, variation in perceptions of quality of care and in rates anxiety and depression due to hospital-level causes could be negligible.

### How do these results compare to other findings?

While rates of anxiety and depression have been shown to vary by individual factors such as younger age (National Breast Cancer Centre and National Cancer Control Initiative 2003), poor social support (National Breast Cancer Centre and National Cancer Control Initiative 2003), cancer type (Zabora *et al*. 2001), advanced disease and the experience of more treatment side effects (National Breast Cancer Centre and National Cancer Control Initiative 2003), to our knowledge, no previous research has examined variation in patient-centred care by hospital setting. Hospital-based variation in other indicators of quality of care suggests that variation in patient perceptions of quality of care should be likely. For example, a study conducted via a population-based cancer registry in the Netherlands identified large variation between sites in patterns of care for lung cancer. Some but not all of this variance was explained by teaching status and patient volume, suggesting the need for further exploration (Elferink *et al*. 2010).

Shultz and colleagues found that timeliness of treatment for lung cancer among veterans was highly variable, with treatment centre characteristics such as treatment in a non-academic clinic, the existence of a specialised diagnostic clinic, leadership beliefs regarding provision of timely treatment and performance of a patient flow analysis associated with a small proportion of the variance (Schultz *et al*. 2009). There is also evidence that system-level factors such as continuity of care (McArdle *et al*. 1996) and mechanisms for encouraging patient questions in medical consultations (Brown *et al*. 2001) do affect cancer patient psychosocial outcomes. Therefore, it is reasonable to consider whether study limitations may underlie the lack of hospital-level variation found in the current study.

### Potential reasons for lack of inter-hospital variation

#### Insufficient variation in the sample of hospitals to detect a difference in outcomes

The present study included only four sites, all of which were large public hospitals in metropolitan New South Wales, Australia. Therefore, it is possible that the characteristics of sites (and providers) which might affect quality of care were very similar in this sample. Therefore, future research should include a larger and more diverse sample of hospitals. However, it should be noted that radiation therapy treatment centres such as those involved in the current study are largely restricted to metropolitan areas of Australia, so a diverse range of patients do attend these centres in order to receive treatment. One of the key challenges for future studies examining the role of hospital variables in determining patient perceptions of quality of care is the need to obtain sufficient data from a broad cross section of patients, providers and sites.

#### Insufficient responsiveness of measures to identify variation between hospitals

The quality of care measure developed for this study has not yet been psychometrically tested. Therefore, it is possible the measure lacked sufficient reliability, sensitivity or breadth to detect differences between hospitals. However, pilot testing of the questions suggested face validity of the items. Furthermore, it is possible that ceiling effects prevented variation in patient views being detected. This explanation does not hold for the failure to detect variation in rates of anxiety and depression using the HADS, given it has been extensively validated in a number of cancer populations (Luckett *et al*. 2010).

## CONCLUSION

In the present study, we examined whether the proportion of patients reporting clinically significant anxiety and depression varied by hospital, and found no significant differences. Similarly, no significant difference was found between the hospitals in the number of domains of quality of care endorsed as needing improvement. This may suggest characteristics of treatment centres such as process of care to not affect psychosocial outcomes. Alternatively, it may suggest that the characteristics of treatment centres in this study were too homogenous for differences in outcomes to be detected. Further research with a larger sample of hospitals will be needed to confirm or refute the present finding. The authors have recently been funded to undertake a larger study examining variation in patient views of quality and psychosocial outcomes across a range of types of hospitals.
